# A thematic analysis of smokers’ and non-smokers’ accounts of E-cigarettes

**DOI:** 10.1177/1359105320909877

**Published:** 2020-03-04

**Authors:** Georgia Louise Wilson, Sarah Grogan, Susan Powell, Ivan Gee, Lorna Porcellato, Joseph Keenan

**Affiliations:** 1 Manchester Metropolitan University, UK; 2 Liverpool John Moores University, UK

**Keywords:** E-cigarettes, inductive thematic analysis, non-smokers, open-ended questionnaire, smokers

## Abstract

This study explored smokers’ and non-smokers’ accounts of E-cigarettes. A total of 51 UK-based participants, 20 men and 31 women, responded to open-ended questions online. Inductive thematic analysis identified that the factors that influence E-cigarette behaviour and opinion in adult smokers and non-smokers are related to *social context, informative sources, practical aspects* and *health implications.* Participants presented varying accounts of E-cigarettes, suggesting that individual narratives regarding E-cigarettes are multi-faceted. This is important information for health professionals and policy makers tasked with advising on E-cigarette use.

The global growth of E-cigarettes (ECs) is an unfolding phenomenon. It is estimated around 3.6 million adults in Great Britain currently use ECs, and there are now more ex-smokers (just under 2 million) using ECs than current smokers (1.4 million; Action on Smoking and Health (ASH), 2019). The increased uptake of ECs among smokers has often been credited to their ability to satisfy nicotine cravings and prevent withdrawal, while also addressing the behavioural-sensory aspects of smoking (Farsalinos, 2018). Around 6.1 per cent of the U.K. population use ECs having never smoked, which is on the increase (ASH, 2019). This presents an emerging demographic of individuals, with new motivations and perceptions which have yet to be explored (Sussan et al., 2017).

Qualitative explorations of EC understanding and behaviour in adult smokers have found a continuum of opinions exist, determined by personal experience and history (Kim et al., 2016; Rooke et al., 2016; Simmons et al., 2016). There is also evidence of uncertainty and misunderstanding regarding the information available surrounding ECs (Vasconcelos and Gilbert, 2018). This is understandable as there is inadequate research and lack of regulatory guidelines combined with an abundance of conflicting information on regulations, brands, flavours, and models (Kaisar et al., 2016).

There is unarguably a lack of qualitative research exploring adult smokers’ and non-smokers’ perceptions of ECs and the factors that may encourage or deter use. There have been few studies focusing on the emerging demographic of EC users who have never smoked cigarettes, and no studies exploring non-smoker attitudes to EC use. Exploring the experience of these individuals is important as there is an increasing demographic that could potentially become addicted to nicotine through a new mode of delivery.

This study provides an opportunity to understand these accounts from the user perspective. It is also of interest to explore non-smokers’ perceptions as non-smoker influence could potentially act as a facilitator and/or barrier in regard to EC use.

## This study

This study set out to examine accounts of ECs from both smokers and non-smokers, as described by the participants themselves, focusing on participants in the U.K. aged between 18 and 65 years. Participants were diverse, and from all genders and all ethnicities. It was important that the participants were English speaking, due to the research relying on qualitative analysis, language and its interpretation.

*Research Question 1.* What are the factors that influence EC behaviour and opinion in adult smokers’ and non-smokers’?

## Method

### Design

To achieve insight into smokers’ and non-smokers accounts of EC use, an open-ended questionnaire (OeQ) design was employed. This qualitative approach provides exploratory information that can attempt to comprehend influencing factors of EC use (Creswell, 2014), including enlightenment on contextual factors and perceptions which may not be captured when using quantitative methods. To encourage disclosure, participants were asked to complete a series of OeQs anonymously online. Pilot work was conducted on an original version of the OeQ. Following the pilot study, the questionnaire received minor amendments to improve clarity.

### Recruitment

Advertisements for the study were placed in suitable locations including EC shops, chemists, libraries, community centres, and University campuses. A snowball sampling approach was also used, and the research team asked their contacts to distribute adverts for the study. The first author also held recruitment events at Manchester Metropolitan University whereby she approached individuals, providing them with the appropriate QR code to access the questionnaire.

### Participants

A total of 51 English speaking respondents, 20 men and 31 women, were recruited. Ages ranged between 18 and 65 years with a mean age of 32.4 years. Table 1 illustrates the demographic characteristics of the participants.

**Table 1.** Demographic characteristics of participants.

Demographic variable	Number of participants	Percentage of participants
Age (in years)		
Mean: 32.4		
Range: 18–65		
Gender		
Male	20	39.22
Female	31	60.78
Ethnicity		
(W) White (Northern Irish/British/Irish)	32	62.74
(M) Mixed/Multiple ethnic groups	2	3.92
(AAB) Asian/Asian British	8	15.69
(BB) Black/African/Caribbean/Black British	0	0
(O) Other Ethnic Group	9	17.65

About 15 participants self-reported successfully quitting smoking using an EC; nine participants self-reported failing to quit smoking using an EC; one participant was a self-reported smoker who also used ECs regularly (dual user); four participants were self-reported smokers who had tried ECs; three participants were self-reported EC users but had never been conventional smokers (identified below using the phrase ‘emerging demographic’); and 19 participants were self-reported never smokers/users. See Supplemental File 1 for full details of participants.

### Materials

An OeQ was constructed guided by previous literature surrounding EC perceptions. The first questions assessed demographic variables such as age, gender, and ethnicity (closed). The participants then answered a question which classified them into one of six categories:

Category 1: I have successfully used an E-cigarette to quit smoking (12 items).
Category 2: I am a smoker who has tried to quit smoking using E-cigarettes but has failed to quit (12 items).
Category 3: I am a smoker who uses E-cigarettes regularly but has no intention to quit (21 items).
Category 4: I am a smoker who has tried an E-cigarette but has no intention to quit (11 items).
Category 5: I have never been a smoker but use E-cigarettes regularly (19 items).
Category 6: I have never smoked conventional cigarettes or used an E-cigarette (12 items).

Each questionnaire contained the same nine general questions (open and closed) which asked about knowledge and opinions of ECs. The general section included OeQs such as ‘what do you think are the positive effects of using E-cigarettes and why?’ Closed ended questions included ‘do you think E-cigarettes are addictive’ with a selection of answers including yes, no, unsure, it depends. The majority of the questions were open. The section also contained some questions with 5-point Likert-type scales whereby participants indicated how much they agree with particular statements such as ‘E-cigarettes encourage non-smokers to start using tobacco cigarettes’. For the purpose of this article which focuses on qualitative responses, only the responses to the (OeQs) are discussed. See Supplement 2 for full list of questions answered by participants in each category.

### Procedure

Ethical approval was first obtained through Manchester Metropolitan University’s ethics committee. The advertisement for the study contained a QR code which took participants to an anonymous *Qualtrics* questionnaire. The advertisement also contained the first author’s email address, to broaden the range of potential participants, that is, those who did not have a device to connect to the Internet when viewing the advertisement. All participants gave informed consent to taking part in the study, including the use of anonymised quotes in reports, through ticking a box on the OeQ to confirm agreement. At the end of the questionnaire, participants were de-briefed and informed of their right to withdraw. They were also given contact details (email) of the researcher for queries or further information regarding the study.

### Data analysis

Braun and Clarke’s (2006) guidelines for inductive thematic analysis were employed to identify themes related to encouraging and deterring EC use, capturing individual understanding and allowing an in-depth analysis of the data. To ensure a respectable analysis the framework recommended by Nowell et al. (2017) was followed which emphasises that interpretivist research is obligated to satisfy the criteria for trustworthiness, which includes credibility (validity); transferability (generalisability); dependability; and confirmability (Lincoln and Guba, 1985; Shenton, 2004). Data were described, summarised, and then interpreted in relation to broader implications.

**Figure 1.**
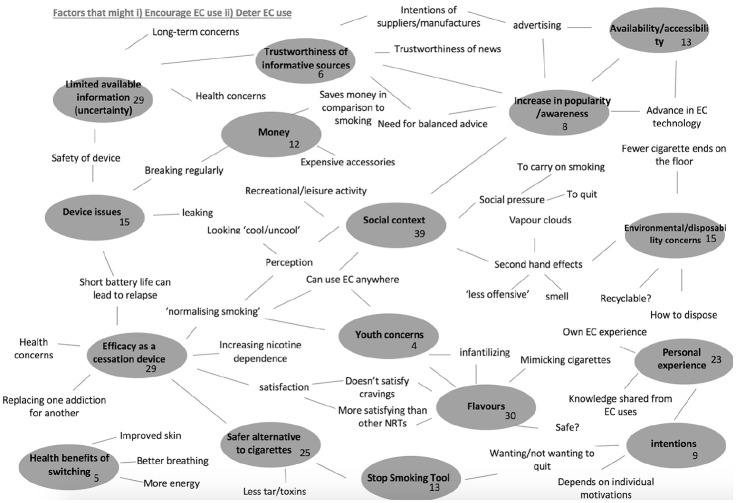
A thematic map illuststrating the subordinate themes and their relationships.

Coding was line by line, allowing data to be organised in to meaningful groups (Tuckett, 2005). Data were initially coded by the first author for content relating to factors that encourage and deter EC use in smokers’ and non-smokers. Descriptive codes based on patterns within the data, were collated with predominant focus on identification of salient themes across the questionnaire responses. These themes were discussed with the second author, revised, and validated by all members of the team. A thematic map of subordinate themes (Figure 1) was generated demonstrating the overall conceptualisation of the data patterns and their relationships (Braun and Clarke, 2006). The research team engaged in reflexive analysis throughout the process of analysing the data following Willig (2008), and all authors agreed thematic structure and content.

## Results and discussion

The analysis identified four key themes evidenced across participant responses. In the quotes below, participants have been given codes to protect their anonymity. An example of an identifying code would be F35W1; this example would denote F (female), aged 35, White and in Category 1.

### Theme 1: social context

Participants noted how vaping acted as a social practice. Those who were part of the emerging demographic (Category 5) embodied this notion of social and recreational vaping claiming they use ECs *‘for fun’* (M18AAB5). Language such as this promotes the image of vaping as a hobby/leisure activity. Placing value on the group experience and social opportunities that come with ECs mirrors previous research (Keane et al., 2016). One participant even expressed the reasons for his EC use in relation to his career:

Just to socialize and sell. If you don’t know anything about a product it’s hard to sell. If you know a lot about a product it is easier to sell. (M19AAB5)

Smoking and EC use appeared to be parallel situational factors that in some cases maintain social connections. For (ex)smokers, ECs could act as an alternative to smoking, though unlike other quit attempts, they do not distance themselves from their existing social networks to avoid relapse. There were contrasting perceptions of ECs linked to social context, with one participant claiming, ‘*a lot of people think its uncool’* (F22O2). Previous research has demonstrated that adult vapers place more value on the group experience and social opportunities that come with ECs (Barbeau et al., 2013; Keane et al., 2016). Therefore, the novelty of vaping could potentially precede and produce a desire to quit smoking, or at least of quitting as a possibility, when previously it may never have been (McNeil, 2015):

A friend recommended me to try it for a week, ever since then I’ve stopped smoking. (M19AAB1)

There was also an indication that how those around them perceived ECs acted as an influencing factor, emphasising the importance of the social context as an encouraging or deterring factor:

When I listen to my family, I guess it is influential in the sense that they recommend the use of e-cigarettes and list the benefits. This is an attempt to convert me (a smoker) to use an e-cigarette – mainly for the health benefits. (F23W4)

Secondhand vapour (SHV) and scent were discussed in relation to social acceptability. Generally, participants believed the vapour from ECs smelt better than conventional tobacco cigarettes (CTC) smoke and for that reason were more socially acceptable:

I think e-cigs are more socially acceptable. As a non-smoker, I have sometimes felt uncomfortable walking past or being near traditional smokers as I really hate the smell and worry that the smell will get onto my clothes and hair, and so I end up worrying about this. (F24W6)

The differences in perception of SHV from ECs in comparison to CTC smoke were sometimes associated with the idea that EC vapour was less damaging and less ‘*irritating for people around me’* (F22O2). However, not all participants agreed with this claiming and there were evident concerns about passive vaping:

Evidently, if e-cigarettes are banned indoors in public places, there must still be concern about secondary smoking effect. (M65O6)

### Theme 2: informative sources

This theme embodies how and where individuals get their knowledge from, the accuracies of this knowledge, how this contributes to attitude, and whether this encourages or deters EC use. There was an element of uncertainty as participants felt that available information on safety is inconsistent:

I have a limited knowledge of the safety of e-cigarettes as there are often conflicting messages in the media. For example, when I first begun using an EC, I read an article that said vaping would lead to ‘popcorn lung’ and could therefore be more harmful than cigarettes. Since then the NHS appears to have supported the use of ECs, this is what led me to try ECs again to reduce the number of cigarettes I use. I don’t know much about the device other than what I have been told in the stores much of my use of ECs is guess work really. (F28W2)

Some participants also expressed scepticism around the sincerity of information sources. Concerns focused on the intention of suppliers and manufactures as it was assumed they are prone to bias and in some cases were thought to have affiliations with the tobacco industry:

. . . the marketing strategies employed by e-cigarette manufactures indicate aggressive efforts to appeal to audiences wider than smokers. I’m suspicious of the manufacturers and suppliers focus on flavour and tastes, as this is of minimal significance to a target population of smokers that have long lost their senses of taste and smell. Granted these senses return and are likely to contribute to their appeal as a cessation aid, but the flavour ranges themselves in many senses are infantilizing. (M28W2)

This has been highlighted as a cause for concern in alternative research (Tamimi, 2017), demonstrating a lack of transparency of manufacturers communications.

Participants across categories were aware that ECs were commonly used as smoking cessation devices to slowly stop smoking. Some also viewed them as a tool to prevent the initial initiation of smoking, that is, for people to use instead of smoking in social situations or to be used by ‘*people that don’t want to start smoking cigarettes*’ (F22O2). Device purpose was therefore understood as a product of individual intentions. Ultimately, whether ECs are viewed as a cessation, recreational or complementary device was seen as depending on the individual and their personal reasons for doing either, echoing concepts from the theory of planned behaviour (TPB; Ajzen, 1991) which proposes that the predominant determinant of individual behaviour is behavioural intention. Examining the intentions of users has proved useful to health care professions, in order to tailor interventions accordingly and provide more customised cessation support to those not satisfied with nicotine replacement therapy (NRT) methods (Wackowski et al., 2016).

Ambivalence was common, although the general consensus across categories was that ECs were better in some senses or ‘the lesser of two evils’ (Shapiro and Kaynar, 2016), though there was a concern that they were not risk free:

I think they are good for heavy smokers who have had difficulty quitting however I think quitting without the use of an E-cigarette would probably be better because I think we are still unsure of what really goes in to an E-cigarette. (F23W1)

As expected, those in Category 6 (non-smokers and non-users) generally claimed to be less knowledgeable about ECs with some claiming they did not know anything about them. There was also a common concern across categories regarding the lack of information about long-term effects of ECs,

At this current moment in time, we do not seem to have steadfast research to suggest the negative effects of vaping, given it is a relatively new idea. I believe there could be extremely negative effects of their use. (M29W1)

These apprehensions reflect past misconceptions in harm reduction strategies such as the ‘light cigarette’ which has led to a mistrust of harm reduction tobacco products (Annechino and Antin, 2019; Farrimond, 2016). Previous research also demonstrates that the lack of reliable information and strong evidence for the effectiveness and safety of ECs acted as a barrier to use (Vasconcelos and Gilbert, 2018).

Personal experience and observations also contributed to how some individuals established their knowledge of ECs. Accessibility was multi-faceted, and ECs were enjoyed due to the convenience of use, being able to use them in a variety of environments including being able to ‘*use them inside*’ (F23W4). This allowed some smokers to regain their freedom as they can be used in a wider variety of places, even where the smoking ban is enforced. However, this raises the concern that it could potentially undermine current tobacco control efforts (Voigt, 2015). There were also concerns that this freedom of use may worsen nicotine dependency:

I think they’re more addictive to e-cigarettes compared to smoking. Because it’s more accessible, doesn’t affect the house/smell bad and it seems less harmful, so I think they ‘vape’ a lot more than they would if they were smoking. This can make them more addicted, or at least more likely to inhale nicotine. My friend has stated that to use cigarettes as a comparison to his e-cigarette habit, he must be smoking the equivalent of 40 a day. It doesn’t stop him though. So, the accessibility and the ‘niceness’ of the e-cigarette, compared to normal cigarettes can make the habit much worse. (F24W6)

Previous research has demonstrated that those who perceive devices as safer alternatives to CTCs, are more likely to distrust healthcare providers, doctors, pharmacists, and other sources (Case et al., 2017). This is an important social risk that should be explored, as it may reveal deeper cultural issues such as the link between the government, public health bodies and the tobacco industry (Tamimi, 2017). Within public health, many harm reduction advocates would argue that the failure to differentiate between industries is a tragedy (Case et al., 2017), as in some cases there are numerous well-meaning EC businesses which have smoking cessation at the centre of their ethos (Ward et al., 2018).

### Theme 3: practical aspects

The third theme focused on practical and physical aspects of EC devices, evidenced by quotes regarding the products and paraphernalia associated with them, combined with the environmental issues that arise from use. It is important to point out that the constituents of this theme were of little relevance to those in Category 6, as participants in this category had no experience using the devices. In regard to smoking cessation attempts, it was common for participants to prefer menthol flavours as these were seen as more closely matching the taste of tobacco cigarettes. E-liquid flavours that could most closely resemble traditional CTCs such as menthol or tobacco appeared to be an encouraging factor for use, particularly among smokers. In the United Kingdom, menthol and tobacco flavours are preferable for those who are attempting to quit smoking (ASH, 2019). Menthol flavours are known to have analgesic and sensory effects which are also present in other tobacco products (Lee and Glantz, 2011), so may somewhat mirror the effects of CTC.

Sweet/fruit flavours such as ‘*cakey*’ (M27W2) and ‘*mango*’ (M28M2) appeared to be common flavours among those had failed to quit smoking using an EC, this may be of significance and could be explored in future research. There were some concerns regarding the safety of the liquids, with one participant saying, ‘*sugary liquids can’t be good for the mouth*’ (M45AAB1).

The potential oral effect of ECs has received surprisingly little attention when considering the intimate relationship of tobacco smoke on oral health, as well as the knowledge that the oral tissues are the first point of contact for EC aerosols when they are at their hottest and most concentrated. One study has found that EC aerosols have similar chemical properties to high-sucrose, gelatinous and acidic drinks (Kim et al., 2018).

Environmental matters were discussed, some participants claimed ECs were better for the environment, with one participant saying, ‘*prevents cigarette butts on the floor which is better for the environment*’ (F24W1). One participant was aware of the appropriate way to dispose of the device parts:

. . . I dispose of my batteries when they no longer hold a charge in a used battery bin. The tank goes in the general waste. (F57W1)

Though some participants seemed less informed:

I would be interested to know how disposable the supposedly disposable cigarettes are as the battery must contain some hazardous waste. (M24W3)

Participants who had used ECs had a better understanding of disposal than non-smokers and non-users, these issues generally did not seem to concern those in Categories 5 and 6. Whether this was encouraging, or deterring was dependent on how they viewed the device in comparison to the damage CTCs have on the environment. There is limited information on the environmental impact of ECs (Chang, 2014). It is vital for public health regulators to maintain that the devices are being disposed of responsibly and ensure the public have access to the knowledge of how to do this so they can make informed decisions.

Practical aspects, such as physical device properties, money and ease of use were important across categories (not including Category 6) when discussing ECs. Device inferiorities were commonly a deterring factor,

. . . not always reliable, high maintenance, not always available as a smoking option. (M21AAB5)

Long battery life was seen as vital and failure in this often led to relapse as participants felt they were ‘*a lot more likely to smoke*’ (M23W1). Device malfunctions were commonly associated with relapse and were a deterring factor as buying replacement parts eventually counteracted the cost-effectiveness of ECs when compared to CTCs. One participant expressed concerns about the device leaking:

I find a lot of ecigs leak which put me off using it. (F24AAB2)

There were also some apprehensions about the safety of the device parts with one participant claiming that poor quality devices could be dangerous, and concerns about batteries which ‘*might explode’* (F19AAB6). A large influencing factor encouraging smokers to try ECs was the amount of money they were spending on CTCs:

The cost for me is the most noticeable positive effect of using an EC. (F28W2)

Previous research has demonstrated that variations in price of devices when compared to combustibles impact the likelihood of smokers switching (Liber et al., 2017). Unfortunately, the aforementioned device inferiorities often led to frequently buying new parts making the cost effectiveness argument unworkable. Experiences of the device as a cessation product was affected by this and differed across categories and, the success rate, shaping the general attitude towards them as a cessation device.

### Theme 4: health implications

The final theme focused on health repercussions, both positive and negative, that arise from EC use regardless of intentions. The efficacy of the device as a cessation method was discussed, understandably those who had managed to quit smoking generally had more positive views of ECs:

Totally effective method that has saved thousands of lives, users are in control of managing their addiction. (M45AAB1)

Successful quit attempts were due to reasons such as assistance in dealing with cravings. It also provided a sense of autonomy as one participant felt in control of managing their addiction. Those who had not managed to quit had more negative perspectives:

E-cigarettes as I see them create a false sense of safety and when coupled with the inability to monitor consumption, a dependency that is difficult to achieve through even the most obscene tobacco use. (M28M2)

Perceived risks play an important role in selecting tobacco products (Hammond and Parkinson, 2009). The Health Belief Model (HBM: Rosenstock, 1974) proposes that perceived risk can affect the motivation to perform a particular health behaviour (Pepper et al., 2015). The degree to which individuals believe ECs to be a less harmful alternative to CTCs will affect the prevalence of their use. Those who initiate EC use for smoking cessation or harm reduction purposes, which are the two most commonly reported goal-orientated reasons (ASH, 2019), may be explicitly or implicitly attempting to reduce their chances of developing a smoking-related illness.

As ECs have rapidly evolved, their nicotine delivery has improved, meaning they may be more attractive to smokers as a replacement (Unger and Unger, 2018). ECs generate an aerosol that penetrates deep into the respiratory tract, which achieves instant absorption of nicotine to the pulmonary venous circulations, mirroring tobacco consumption in the form of CTCs (Sosnowski and Odziomek, 2018). Although this could be viewed positively, this means that the addiction potential has also increased (Unger and Unger, 2018). Concerns about nicotine dependency were also a deterring factor; once participant suggested the devices should have specific mechanism in order to prevent nicotine abuse:

An automatic locking mechanism that prevents nicotine flooding/abuse. My latest e-cigarette had such a feature. (M28W1)

Even for those who had manage to quit smoking CTCs there were still hesitations about the device as replacing cigarette addiction with an EC addiction:

I have given up cigarettes but just adopted another addiction with E-cigarettes although it is not as bad as cigarettes, I don’t think I could ever quit both. (F22O1)

Smokers and ex-smokers noticed differences from switching from CTCs to ECs including improvements in skin, breathing, and energy. Although noticeably beneficial for some, not all participants were convinced:

A small number of benefits come to mind, but they’re outweighed by the negatives. (M28M2)

It is important to examine the roles of affect and perceived risks in tobacco and nicotine products (Popova et al., 2018). Research should ensure to differentiate between perceived risk and actual negative experience. Given the lack of scientific agreement and uncertainty surrounding the use of devices, means to clearly convey accurate information need to be considered.

### Strengths and limitations

Strengths of this study were that participants were detailed in their responses and shared a large amount of information. The anonymity of the process enabled this level of disclosure. This study also broadened the participant pool by accessing participants from a range of ages and genders. There were undeniable limitations to the study. Accounts are from participants from one geographical area of the United Kingdom so it is uncertain how far these responses would generalise to others outside this area. There are disadvantages to the snowball sampling strategy, as it is not random and can therefore lead to bias (Emerson, 2015). Socioeconomic status (SES) was also not explored in this study, which is limiting, as previous research has demonstrated it has been linked to differences in perceptions of ECs (Hartwell et al., 2017). Future research could compare themes between people in different SES groups. There were also conceptual challenges such as self-categorisation of smoking/EC use; there is a possibility that participants could self-identify incorrectly due to social expectations. Although participants in this study varied in ethnicity, participants largely identified as white, so it is uncertain how far these responses can generalise to other ethnicities. Further research could examine this through a wider group of participants with varied ethnicities, as well as from wider range of geographical areas.

### Key implications

1. Future research should continue to explore the social practice, including perceptions of SHV that surround vaping behaviour to provide more effective ways of understanding and conceptualising attitudes towards ECs, as well shifting the focus from individuals as the agent of behaviour, towards alliances between EC behaviour and social practices.
2. There is a need for more transparency between communication systems. It is important for information that is available to be accurate and communicated efficiently to avoid stigmatising ECs, which could prevent smokers from wanting to use them, while also ensuring non-smokers are deterred from using them. Harm-reduction campaigns should ensure that it is clear when information comes from credible sources or is a form of marketing, in order to accurately influence EC attitudes and knowledge.
3. It is important to find a balance between cost efficiency without compensating for device product quality, while also ensuring the cost is high enough to deter youth access. The most cost-effective method for cessation is important for public health gain. The accessibility of EC products highlights an important risk factor of smoking relapse. Future harm reduction policies should consider this and contemplate implementing policies to allow EC paraphernalia to be more readily available than CTCs. It is also vital for waste disposal authorities to maintain that the devices are being disposed of responsibly and ensure the public have access to the knowledge of how to do this, so they can make informed decisions. Health policy debates around ECs should consider the health of the environment.
4. Given the general misunderstanding on the health effects of ECs and the vital role of perception in behaviour, health care providers, health education practitioners, campaign designers and policy makers should remain vigilant and unbiased when advising on ECs.

### Reflexive analysis

We have tried to present participant accounts fairly and disinterestedly. The first author is a Ph.D. student in psychology, and the other authors are the supervisory team which consisted of five academics, two from health psychology and three from public health. The analysis benefits from having a range of perspectives on ECs from those in a range of disciplines. However, our roles as academics involved in health promotion may have influenced our analysis of data, so this needs to be taken into account when reading our analysis and interpretation.

## Conclusion

This study demonstrates the variability of EC experiences. The social context surrounding individuals moulds their experience and perception of ECs. The intentions and implications of informative sources absorbed also shape individual accounts. Practical and physical aspects of EC devices, and how users and non-users have experienced these individually, contributes towards their perception. The health implications of ECs highlight both the positive and negative effects of ECs. It is therefore important that health professionals do not expect homogeneous patterns of experiences, so tailored and efficient advice can be given.

## Supplemental Material

E-cigarettes_Supplemental_File_1 – Supplemental material for A thematic analysis of smokers’ and non-smokers’ accounts of E-cigarettes Click here for additional data file. Supplemental material, E-cigarettes_Supplemental_File_1 for A thematic analysis of smokers’ and non-smokers’ accounts of E-cigarettes by Georgia Louise Wilson, Sarah Grogan, Susan Powell, Ivan Gee, Lorna Porcellato and Joseph Keenan in Journal of Health Psychology

E-cigarettes_Supplemental_File_2 – Supplemental material for A thematic analysis of smokers’ and non-smokers’ accounts of E-cigarettes Click here for additional data file. Supplemental material, E-cigarettes_Supplemental_File_2 for A thematic analysis of smokers’ and non-smokers’ accounts of E-cigarettes by Georgia Louise Wilson, Sarah Grogan, Susan Powell, Ivan Gee, Lorna Porcellato and Joseph Keenan in Journal of Health Psychology
