# Supplementary material for: A thematic analysis of smokers’ and non-smokers’ accounts of E-cigarettes
Source: J Health Psychol. 2020 Mar 4;27(1):24–35. doi: 10.1177/1359105320909877 (PMC8739557; doi:10.1177/1359105320909877)
Supplement: E-cigarettes_Supplemental_File_1 – Supplemental material for A thematic analysis of smokers’ and non-smokers’ accounts of E-cigarettes [file E-cigarettes_Supplemental_File_1.pdf]

Supplement 1: Participant Demographics and Category

No	Gender	Age	Ethnicity	Category
1	Male	36	Other: White other	Successfully quit smoking using an E-cigarette (1)
2	Male	45	Asian/Asian British	Successfully quit smoking using E-cigarette (1)
3	Female	28	White (Northern Irish/British/Irish)	Failed to quit smoking using an E-cigarette (2)
4	Female	23	White (Northern Irish/British/Irish)	Successfully quit smoking using an E-cigarette (1)
5	Female	22	White (Northern Irish/British/Irish)	I have never smoked conventional cigarettes of used an E-cigarettes (6)
6	Female	19	White (Northern Irish/British/Irish)	Successfully quit smoking using an E-cigarette (1)
7	Male	19	White (Northern Irish/British/Irish)	Successfully quit smoking using an E-cigarette (1)
8	Female	24	Asian/Asian British	Failed to quit smoking using an E-cigarette (2)
9	Male	26	Other: Lithuanian	Failed to quit smoking using an E-cigarette (2)
10	Female	35	White (Northern Irish/British/Irish)	Successfully quit smoking using an E-cigarette (1)
11	Female	22	Other: Arab	Failed to quit smoking using an E-cigarette (2)
12	Female	18	White (Northern Irish/British/Irish)	I have never smoked conventional cigarettes or used an E-cigarette (6)
13	Male	19	Asian/Asian British	Successfully quit smoking using an E-cigarette (1)
14	Male	25	White (Northern Irish/British/Irish)	Failed to quit smoking using an E-cigarette (2)
15	Female	19	Asian/Asian British	I have never smoked conventional cigarettes or used an E-cigarette (6)
16	Female	25	Other: Arab	I have never smoked conventional cigarettes or used an E-cigarette (6)
17	Male	21	Other: Pakistani	I have never been a conventional cigarette smoked but I use E-cigarettes regularly (5)
18	Male	27	White (Northern Irish/British/Irish)	Failed to quit smoking using an E-cigarette (2)
19	Male	18	Asian/Asian British	I have never been a conventional cigarette smoked but I use E-cigarettes regularly (5)
20	Female	34	Asian/Asian British	I have never smoked conventional cigarettes or used an E-cigarette (6)
21	Female	23	White (Northern Irish/British/Irish)	I am a smoked who has tried E-cigarettes but has no intention to quit (4)
22	Male	24	White (Northern Irish/British/Irish)	I am a smoked who has tried E-cigarettes but has no intention to quit (4)
23	Male	N/A	White (Northern Irish/British/Irish)	I am a smoked who uses E-cigarettes but has no intention to quit (3)
24	Female	22	Other: Arab	Failed to quit smoking using an E-cigarette (2)

25	Male	23	Asian/Asian British	Failed to quit smoking using an E-cigarette (2)
26	Male	19	Asian/Asian British	I have never been a conventional cigarette smoked but I use E-cigarettes regularly (5)
27	Male	28	Mixed/Multiple ethnic groups	Failed to quit smoking using an E-cigarette (2)
28	Female	53	White (Northern Irish/British/Irish)	I have never smoked conventional cigarettes or used an E-cigarette (6)
29	Female	56	White (Northern Irish/British/Irish)	I have never smoked conventional cigarettes or used an E-cigarette (6)
30	Female	24	White (Northern Irish/British/Irish)	I have never smoked conventional cigarettes or used an E-cigarette (6)
31	Male	29	White (Northern Irish/British/Irish)	Successfully quit smoking using an E-cigarette (1)
32	Female	35	White (Northern Irish/British/Irish)	Successfully quit smoking using an E-cigarette (1)
33	Male	54	White (Northern Irish/British/Irish)	I have never smoked conventional cigarettes or used an E-cigarette (6)
34	Female	22	White (Northern Irish/British/Irish)	I have never smoked conventional cigarettes or used an E-cigarette (6)
35	Male	28	Other: Greek	Successfully quit smoking using an E-cigarette (1)
36	Female	32	Other: Other white	I have never smoked conventional cigarettes or used an E-cigarette (6)
37	Female	46	White (Northern Irish/British/Irish)	I have never smoked conventional cigarettes or used an E-cigarette (6)
38	Female	48	White (Northern Irish/British/Irish)	I have never smoked conventional cigarettes or used an E-cigarette (6)
39	Female	60	White (Northern Irish/British/Irish)	I have never smoked conventional cigarettes or used an E-cigarette (6)
40	Female	20	White (Northern Irish/British/Irish)	I am a smoked who has tried E-cigarettes but has no intention to quit (4)
41	Female	24	White (Northern Irish/British/Irish)	I have never smoked conventional cigarettes or used an E-cigarette (6)
42	Male	65	Other: Mediterranean	I have never smoked conventional cigarettes or used an E-cigarette (6)
43	Female	40	Mixed/Multiple ethnic groups	I have never smoked conventional cigarettes or used an E-cigarette (6)
44	Female	60	White (Northern Irish/British/Irish)	Successfully quit smoking using an E-cigarette (1)
45	Male	29	White (Northern Irish/British/Irish)	I have never smoked conventional cigarettes or used an E-cigarette (6)
46	Female	24	White (Northern Irish/British/Irish)	Successfully quit smoking using an E-cigarette (1)
47	Female	57	White (Northern Irish/British/Irish)	Successfully quit smoking using an E-cigarette (1)
48	Female	55	White (Northern Irish/British/Irish)	Successfully quit smoking using an E-cigarette (1)
49	Female	21	White (Northern Irish/British/Irish)	I am a smoked who has tried E-cigarettes but has no intention to quit (4)

50	Female	46	White (Northern Irish/British/Irish)	I have never smoked conventional cigarettes or used an E-cigarette (6)
51	Male	43	White (Northern Irish/British/Irish)	Successfully quit smoking using an E-cigarette (1)
