# Supplementary material for: A thematic analysis of smokers’ and non-smokers’ accounts of E-cigarettes
Source: J Health Psychol. 2020 Mar 4;27(1):24–35. doi: 10.1177/1359105320909877 (PMC8739557; doi:10.1177/1359105320909877)
Supplement: E-cigarettes_Supplemental_File_2 – Supplemental material for A thematic analysis of smokers’ and non-smokers’ accounts of E-cigarettes [file E-cigarettes_Supplemental_File_2.pdf]

Supplement 2: Open-ended questions and Relating Participant Categories

Question	Participant Category
**What do you know about E-cigarettes (what are they used for, how do they work, types of devices, disposability, liquids available, etc.)? Please provide as much detail as possible**	1,2,3,4,5,6
(Following a closed-ended question asking: Where would you say you have acquired the majority of your knowledge about E-cigarettes? **Out of the options you have selected please discuss which has been the most influential and why you think this is in as much detail as possible**	1,2,3,4,5,6
**What do you think are the positive effects of using an E-cigarettes and why? Please provide as many examples as you can**	1,2,3,4,5,6
**What do you think are the negative effects of using an E-cigarettes and why? Please provide as many examples as you can**	1,2,3,4,5,6
(Following closed question: What do you think are the main motivations for an individual to begin using E-cigarettes? Please select all that apply **Out of the options you have selected, please select which you think is the most important and discuss why in as much detail as possible**	1,2,3,4,5,6
**What are your opinions on the use of E-cigarettes to help people to stop smoking? Please provide as much detail as possible**	1,2,3,4,5,6
(Closed question: do you think e-cigarettes are addictive?) **Please explain in as much detail as possible your reasons behind this**	1,2,3,4,5,6
(Closed question: On a scale of 1-5 (1 = Strongly Disagree, 5 = Strongly Agree) please rate how much you agree with the following statement by selecting the appropriate box: ‘E-cigarettes encourage non-smokers to start using tobacco cigarettes’ **Please discuss the reasons behind your rating in as much detail as possible**	1,2,3,4,5,6
**The legal age of sale for E-cigarettes in the United Kingdom is 18 years of age, what is your opinion regarding this? Please provide as much detail as possible**	1,2,3,4,5,6
**What do you want to know about E-cigarettes?**	
**Which aspects of E-cigarettes do you think requires more research?**	
**Please describe the details of your tobacco use before you started using an E-cigarette (i.e. how often did you smoke, when did you start smoking, how long did you smoke for, how much you spent on tobacco, why did you want to quit, etc.?) in as much detail as possible**	1,2,3
Closed question: Earlier, you discussed the main motivations for any given individual to begin using an E-cigarette: what were *your* main motivations for beginning to use an E-cigarette? Please select all that apply)	1,2,3

**Out of the options you have selected, please select which you think is the most important and discuss why in as much detail as possible**	
**Please discuss in as much detail as possible your first E-cigarette experience, exploring to how this has changed overtime with reference to the generation, the brand the flavour liquid, the nicotine content, etc.**	1,2,3
**Are there any characteristics that are important to you when purchasing an E-cigarette that have not been mentioned?**	1,2,3
**Out of the characteristics you have selected, please discuss the characteristic(s) you have rated the highest and why in as much detail as possible**	1,2,3
**In regard to e-liquid flavours, what are your preferences and why?**	1,2,3,5
**Please describe the details of where you purchase your E-cigarette products and the reasons behind this**	1,2,3
**You have successfully used an E-cigarette to quit smoking: please provide a detailed step-by-step description in as much detail as possible of how you did this (including any details of other forms of quitting support), and why you think you were able to quit**	1
**Is there anything you could suggest from your experience that could make E-cigarettes more efficient in helping users quit conventional smoking?**	1,2,3
**Is there anything else you would like to add?**	1,2,3,4,5,6
**As you claimed, you failed to quit smoking using an E-cigarette: please provide a step-by-step description in as much detail as possible of how you did this (including any details of other forms of quitting support), and why you think you were unable to quit**	2
**Please explain in as much detail as possible why you smoke conventional cigarettes and use an E-cigarette rather than using just one or the other**	3
**Why did you start using an E-cigarette?**	5
**Based on your experience is there anything you could suggest that would prevent non-smokers trying e-cigarettes?**	5
**Why have you never smoked tobacco cigarettes?**	6
**Why have you never used an E-cigarette**	6
